# Dexamethasone does not prevent hydrocephalus after severe intraventricular hemorrhage in newborn rats

**DOI:** 10.1371/journal.pone.0206306

**Published:** 2018-10-25

**Authors:** Jang Hoon Lee, Yun Sil Chang, So Yoon Ahn, Se In Sung, Won Soon Park

**Affiliations:** 1 Department of Pediatrics, Ajou University School of Medicine, Suwon, Republic of Korea; 2 Department of Pediatrics, Samsung Medical Center, Sungkyunkwan University School of Medicine, Seoul, Republic of Korea; 3 Stem Cell and Regenerative Medicine Institute, Samsung Medical Center, Seoul, South Korea; 4 Department of Health Sciences and Technology, SAIHST, Sungkyunkwan University, Seoul, South Korea; Loma Linda University School of Medicine, UNITED STATES

## Abstract

The aim of this study was done to determine whether dexamethasone treatment prevents posthemorrhagic hydrocephalus (PHH) development and attenuates brain damage after severe IVH in newborn rats. Severe IVH was induced by injecting; 100 μL of blood into each lateral ventricle of postnatal day 4 (P4) Sprague-Dawley rats. Dexamethasone was injected intraperitoneally into rat pups at a dose of 0.5 mg/kg, 0.3 mg/kg, and 0.1 mg/kg on P5, P6, and P7, respectively. Serial brain magnetic resonance imaging and behavioral function tests, such as the negative geotaxis test and the rotarod test, were performed. On P32, brain tissues were obtained for histological and biochemical analyses. Dexamethasone treatment significantly improved the severe IVH-induced increase in the terminal deoxynucleotidyl transferase-mediated deoxyuridine triphosphate nick end-labeling-positive cells, glial fibrillary acidic protein-positive astrocytes and ED-1 positive microglia, and the decrease in myelin basic protein. IVH reduced a survival of 71%, that showed a tendency to improve to 86% with dexamethasone treatment, although the result was not statistically significant. However, dexamethasone failed to prevent the progression to PHH and did not significantly improve impaired behavioral tests. Similarly, dexamethasone did not decrease the level of inflammatory cytokines such as interleukin (IL) -1α and ß, IL-6, and tumor necrosis factor-α after severe IVH. Despite its some neuroprotective effects, dexamethasone failed to improve the progress of PHH and impaired behavioral tests after severe IVH.

## Introduction

Intraventricular hemorrhage (IVH) results from the rupture of the germinal matrix hemorrhage through the ependymal into the lateral ventricle and is a common and serious disorder in premature infants [1, 2]. Premature infants with severe IVH have a high risk of brain damage and progression to posthemorrhagic hydrocephalus (PHH), which can result in increased mortality and neurologic morbidities such as seizure, cerebral palsy, and developmental retardation in survivors [3–5].

Although the pathogenesis of brain damage and the progression to PHH from severe IVH has not been completely delineated, it is known that the contact of blood and blood products in the subarachnoid space leads to an inflammatory response that can cause obliterative arachnoiditis. This condition can cause dysfunction in arachnoid granulations, which can reduce cerebrospinal fluid (CSF) resorption and increase intracranial pressure, resulting in a venous infarction with reduced cerebral perfusion [6–8]. Furthermore, inflammatory cytokines from blood products in the ventricles may injure the periventricular white matter [7, 9]. Currently, no effective treatment is clinically available to attenuate brain injury and prevent progression to PHH after severe IVH in premature infants. Therefore, new and effective therapeutic modalities with strong anti-inflammatory capabilities to treat brain injury and the progression to PHH after severe IVH would be of great value.

Glucocorticoids such as dexamethasone are one of the most potent anti-inflammatory agents owing to their ability to suppress many inflammatory indices [10]. They are currently used to treat chronic inflammatory diseases such as asthma [11] or as adjuvant therapy to improve the clinical outcome of bacterial meningitis in children [12]. Glucocorticoids are also widely used in perinatal and neonatal medicine. Antenatal administration of glucocorticoids before 34 weeks of gestation improved the composite outcomes of mortality and major morbidities including severe IVH, necrotizing enterocolitis and bronchopulmonary dysplasia (BPD) [13]. However, repeated treatment with antenatal glucocorticoids might reduce fetal brain growth and impair long-term neurodevelopmental outcomes [14]. Postnatal glucocorticoid use in premature infants to prevent or treat BPD might increase the risk of neurodevelopmental impairment and cerebral palsy, particularly in the case of dexamethasone treatment within the first week of life [15]. Furthermore, the available data on the therapeutic efficacy of glucocorticoids for treating brain hemorrhages studies remain largely controversial [16–21]. Therefore, further studies are necessary to clarify the role of glucocorticoid treatment for severe IVH in premature infants. In this study, we investigated whether dexamethasone treatment could attenuate brain damage and the progression to PHH after severe IVH in newborn rats.

## Materials and methods

### Animal model

All works regarding animal procedure described herein were reviewed and approved by the Animal Care and Use Committee (ACUC) of Samsung Biomedical Research Institute (SBRI), Seoul, Republic of Korea (approval number: 20141125003), and was conducted in accordance with the Institutional and National Institutes of Health Guidelines for Laboratory Animal Care. The ACUC of SBRI specifically reviewed and approved the anticipated mortality in the study design. All animal procedures were done in an Association for Assessment and Accreditation of Laboratory Animal Care-accredited specific pathogen-free facility. All the investigators who performed the animal experiment in this study received the education course for animal care conducted by the ACUC of SBRI.

**Fig 1:**
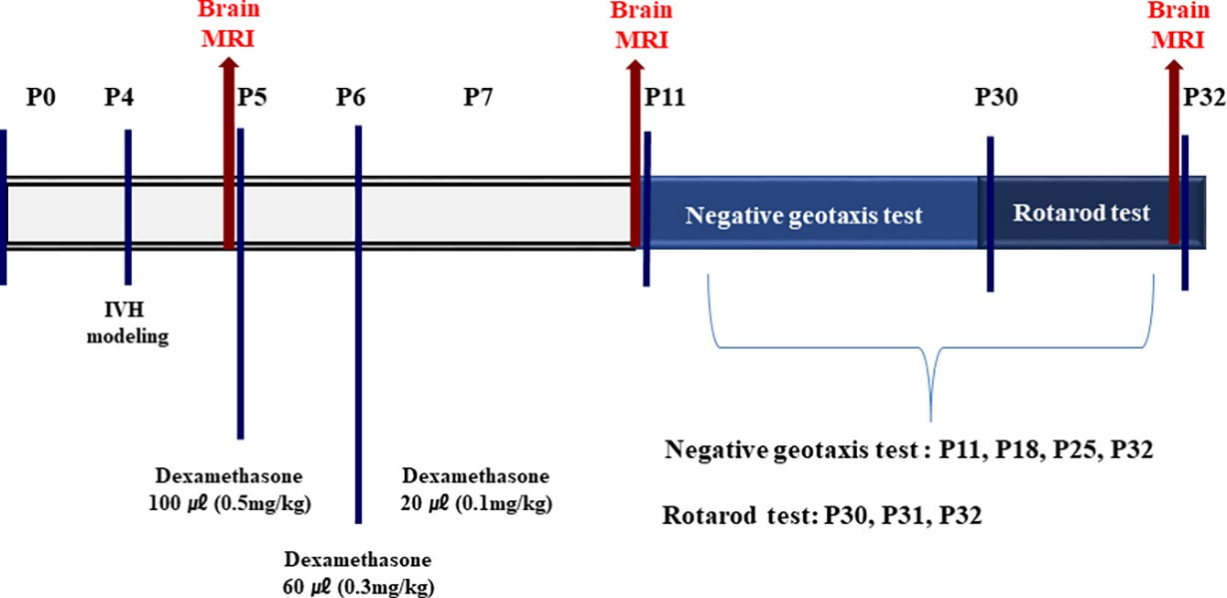
Experimental protocol. MRI refers to magnetic resonance imaging and IVH to intraventricular hemorrhage. Dexamethasone was injected intraperitoneally.

Newborn male Sprague-Dawley rat pups (Orient Bio Co., Seoul, Republic of Korea) were reared with their dams. Only male rat pups were used in this study to exclude any gender-related differences in brain injury [22]. Fig 1 shows details of the study protocols. The experiment started four days postnatal (P4) and ended on P32. To induce severe IVH, the P4 rat pups that were selected with a table of random unit, were anesthetized using 1.5% to 2% isoflurane in oxygen-enriched air, and a 200 μL of fresh maternal whole blood was slowly infused into the right and left ventricles (100 μL into each ventricle) under stereotactic guidance (Digital Stereotaxic Instrument with Fine Drive, MyNeurolab, St. Louis, MO; coordinates: *x* = ±0.5, *y* = +1.0, *z* = +2.5 mm relative to the bregma) [23]. There was no mortality associated with the IVH induction. The normal control group (NC, n = 9) underwent a sham operation without blood injection. After the induction of IVH, the rat pups were allowed to recover; and were returned to their dams. On P5, severe IVH induction was confirmed via brain magnetic resonance imaging (MRI), and the rat pups were randomly divided into an IVH with dexamethasone treatment group (ID, n = 21), and an IVH control group (IC, n = 21). The ID group was treated with intraperitoneal injections of dexamethasone at doses of 0.5 mg/kg (100 μL), 0.3 mg/kg (60 μL), and 0.1 mg/kg (20 μL) on P5, P6, and P7, respectively. Pups in the IC group were given an equal volume of normal saline on P5, P6, and P7. Follow-up brain MRIs were performed on P11 and P32. Negative geotaxis tests were performed on P11, P18, P25, and P32, and rotarod test were done from P30 to P32. All rat pups were weighed daily and euthanized on P32 under deep pentobarbital anesthesia (60 mg/kg, intraperitoneal route). All experimental works inducing pain were done under the isoflurane inhaled anesthesia. All animals’ condition and mortality were monitored on a daily basis.

### Brain MRI and assessment of the ventricle-to-whole-brain ratio

Brain MRIs were performed on neonatal rat pups to confirm the successful induction of severe IVH on P5 and subsequently to monitor on P11 and P32. The MRIs were performed by an investigator blinded to the treatment groups. MRIs were performed using a 7.0-Tesla MRI system (Bruker-Biospin, Fällanden, Switzerland) as described in a previous study [24]. The ventricle-to-whole-brain volume ratio was calculated for each pup. Two blinded investigators independently calculated the volume ratios by manually outlining the ventricle and the whole brain referencing 12 MRI slices with the ParaVision software (version 2.0.2, Bruker, BioSpin, Karlsruhe, Germany) as previously described [23, 24]. Cavalieri’s principal was employed to estimate the brain volume [24] and the ventricle-to-whole-brain volume ratio was calculated to determine the extent of PHH after IVH induction.

### Behavioral function tests

Negative geotaxis tests were conducted on P11, P18, P25, and P32 [24]. We recorded the time it took for the pups to rotate 180° to face uphill after they were released on a slanted slope. The average daily time of three trials was used for subsequent analysis. Three consecutive rotarod tests were performed from P30 to P32 to assess motor function by analyzing the latency to fall on a treadmill test [25, 26]. Each day, all rat pups were tested three times consecutively with a 15-minute break between trials. The average of the three trials was used as the final latency result for that day. All behavioral function tests were conducted independently by two examiners who were blind to the group assignments.

### Terminal deoxynucleotidyl transferase dUTP nick end labeling (TUNEL) assay

Cell death in the periventricular white matter was assessed via TUNEL assay (kit S7110 ApopTag, Chemicon, Temecula, CA). An investigator blind to the group assignments counted the TUNEL-positive nuclei in the periventricular area on coronal brain sections, including the corpus callosum and caudate nucleus. TUNEL-positive nuclei were also counted from three random non-overlapping fields of three coronal sections (+0.95 mm to −0.11 mm/Bregma) from each brain.

### Immunohistochemistry

Immunohistochemical analyses of gliosis (neuronal specific glial fibrillary acidic protein [GFAP]), myelination (myelin basic protein [MBP]), and reactive microglia (ED-1), were performed on deparaffinized 4-μm thick brain sections, as described in a previous study [24]. The immunofluorescent GFAP or MBP staining intensity was measured in three random non-overlapping fields of the corpus callosum on three coronal sections (+0.95 mm to −0.11 mm/Bregma) by a blinded investigator using Image J software (National Institute of Health).

### Enzyme-linked immunosorbent assay (ELISA)

The concentrations of interleukin (IL)-1α, IL-1β, IL-6, and tumor necrosis factor (TNF)-α in brain tissue homogenates were measured using a Milliplex MAP ELISA Kit according to the manufacturer’s protocol (Millipore, Billerica, MA).

### Statistical analyses

Data are expressed as the mean ± SEM. For continuous variables with a normal distribution, statistical comparisons among the three groups were performed using a one-way analysis of variance (ANOVA) with a Bonferroni correction. *P* < .05 was considered statistically significant. Stata software (version 11.0, Stata Corp LP, College Station, TX) was used for the analyses.

## Results

### Survival and body weight

**Fig 2:**
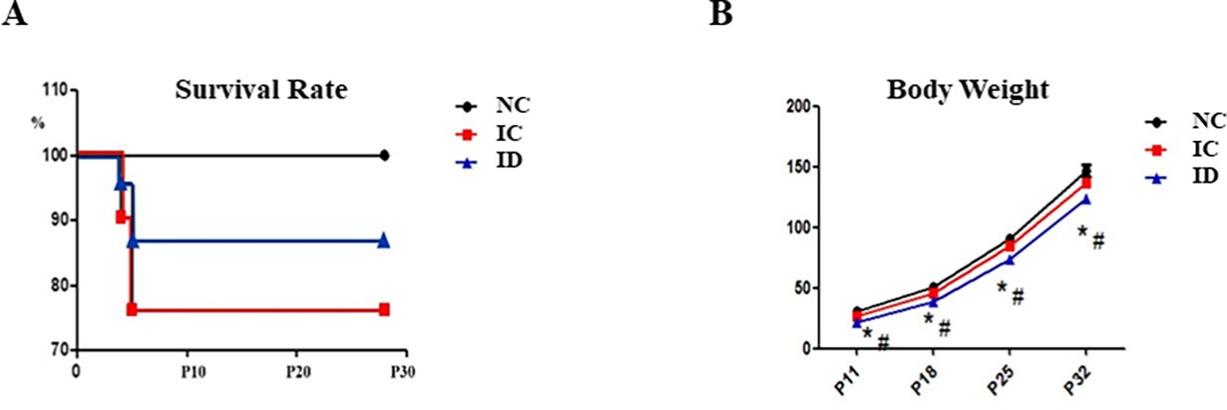
Survival rates and changes in body weight. There were no significant differences in survival among the groups. The survival rate of the IVH + dexamethasone (ID, n = 21), the IVH control (n = 21), and the normal control (NC, n = 9) were 86%, 71%, and 100%, respectively (A). ID group (n = 18) group had the smallest weight gain between P11 to P32. NC group (n = 9), IC group (n = 15) (B). IVH refers to intraventricular hemorrhage. *P < .05 compared to NC group. # *P* < .05 compared to IC group.

There were no significant differences in survival between the groups: 9 of 9 (100%), 15 of 21 (71%), and 18 of 21(86%) for the NC, IC, and ID groups, respectively (Fig 2A). The ID group had the least weight gain between P11 and P32, and the final weights on P32 were 147 ± 5 g, 137 ± 3 g, and 124 ± 3 g, for the NC, IC, and ID groups, respectively (*P* < .05) (Fig 2B).

### Serial brain MRI

**Fig 3:**
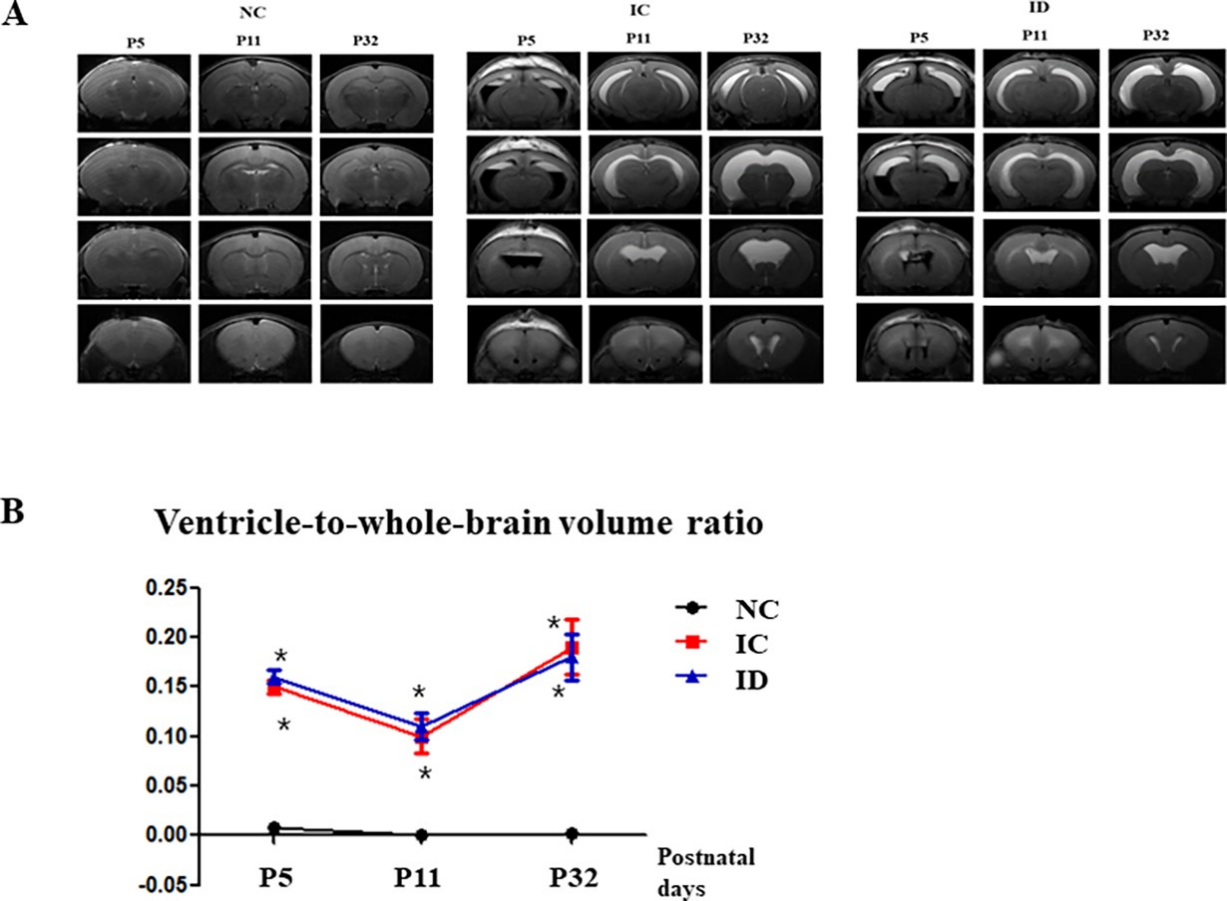
Postnatal dexamethasone did not attenuate ventricular dilatation after induced IVH. **(A) Brain MRI serials of the three groups. (B) Ventricle-to-whole-brain volume ratio**. The numbers are expressed as the mean ± SEM. MRI refers to magnetic resonance imaging. IVH refers to intraventricular hemorrhage. *P < .05 compared to the normal control (NC, n = 9). IC, IVH control (n = 20); ID, IVH + dexamethasone (n = 21).

Ventricles dilated and filled with blood from induced IVH were confirmed by brain MRIs on P5, P11, and P32. Fig 3A shows serial brain MRIs of all groups at each time point (P5, P11, and P32). The ventricle-to-whole-brain volume ratios reflect the severity of ventricular dilatation, and were significantly lower in the NC group than in the IC and ID groups (0.0 ± 0.0%, 15.6 ± 0.6%, 16.3 ± 0.7% on P5, 0.0 ± 0.0%, 10.5 ± 1.7%, 10.9 ± 1.4% on P11, and 0.0 ± 0.0%, 19.6 ± 2.8%, 17.6 ± 2.7% on P32, respectively (all *P <* .05)). However, this ratio did not differ significantly between the IC and ID groups on P5, P11, or P32 (*P* > .05) (Fig 3B).

### Behavioral function tests

**Fig 4:**
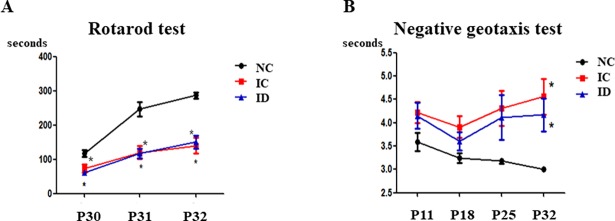
Sensorimotor functions did not improve after dexamethasone treatment. The outcomes of sensorimotor functions via rotarod test (A) and negative geotaxis test (B). The numbers are expressed as the mean ± SEM. IVH refers to intraventricular hemorrhage. *P < .05 compared to the normal control (NC, n = 9). IC, IVH control (n = 15); ID, IVH + dexamethasone (n = 18).

The rotarod test and the negative geotaxis test were used to evaluate sensorimotor functions. The rotarod test was conducted on P30, P31, and P32. The NC group displayed a longer latency to falling than the IC and ID groups (*P* < .05) but no difference was observed between the IC and ID groups (Fig 4A). The negative geotaxis test was performed on P11, P18, P25, and P32. Although no significant differences were found among the three groups on P11, P18, and P25, the NC group responded more quickly than the IC and ID groups on P32 (3.0 ± 0.0, 4.6 ± 0.4, and 4.2 ± 0.4, respectively (*P* < .05) (Fig 4B).

### Cell death, reactive gliosis, and myelination

**Fig 5:**
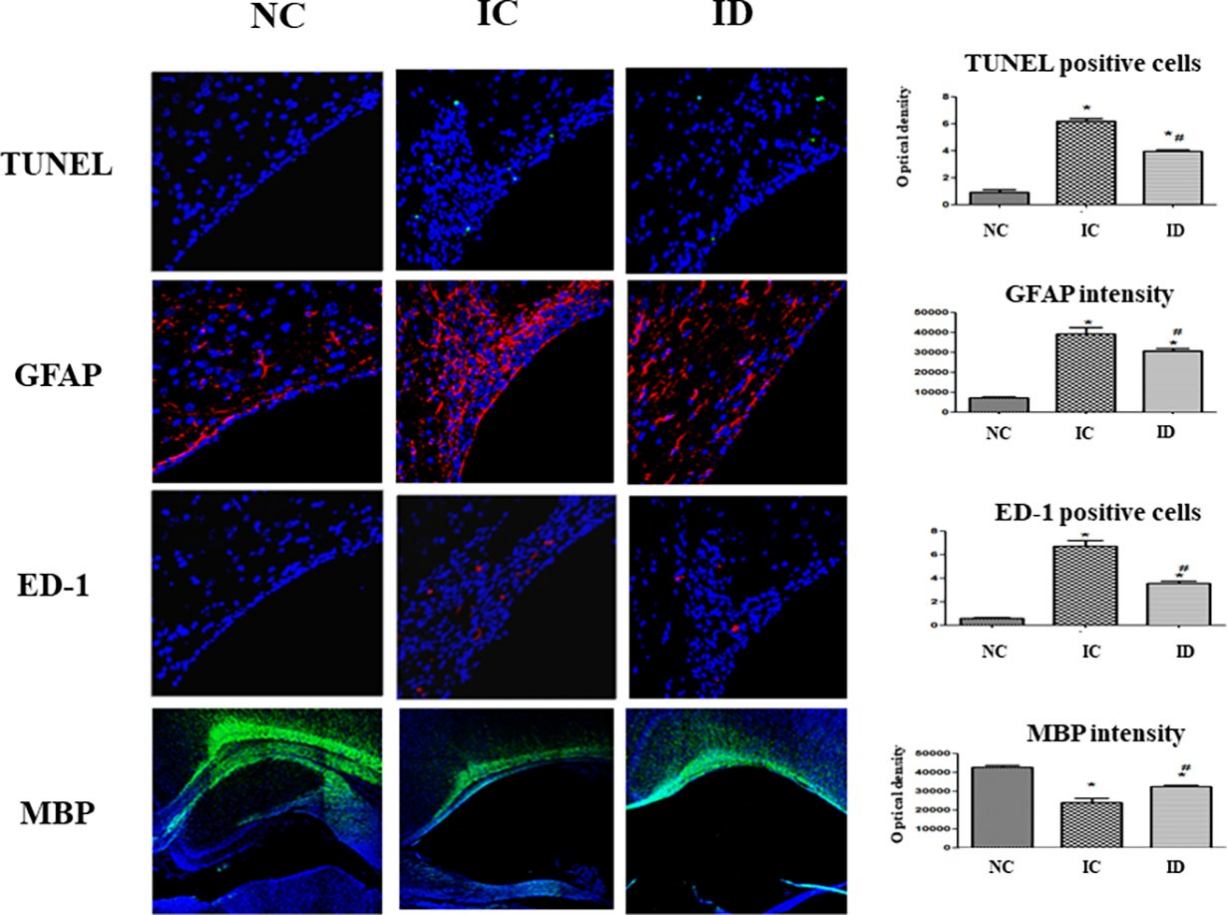
Cell death and reactive gliosis induced by severe IVH showed a decrease after dexamethasone treatment. Representative immunofluorescence photomicrographs of the periventricular area with staining for terminal deoxynucleotidyl transferase nick-end labeling (TUNEL, green), glial fibrillary acidic protein (GFAP, red), ED-1 (red), and myelin basic protein (MBP, green, original magnification ×400; scale bars = 25 μm). The average number of TUNEL-positive cells, the average density of GFAP staining, the average number of ED-1-positive cells, and the average MBP density in the periventricular area are shown. The numbers are expressed as the mean ± SEM. IVH refers to intraventricular hemorrhage. *P < .05 compared to the normal control (NC, n = 4) group, # *P* < .05 compared to the IVH control (IC, n = 5). ID, IVH + dexamethasone (n = 6).

Immunohistochemistry analysis was used to determine whether dexamethasone attenuated periventricular cell death and reactive gliosis after severe IVH. The number of TUNEL-positive cells and the density of GFAP-positive cells in the periventricular area were assessed in brain tissue on P32. The number of TUNEL-positive cells and the density of GFAP-positive cells in the IC group was significantly higher than in the NC group, which indicated that dexamethasone significantly ameliorated cell death and the reactive gliosis in periventricular brain tissue after severe IVH. The number of macrophages (ED-1–positive cells) in the periventricular area assessed on P32 was significantly greater in the IC group than the NC group, indicating dexamethasone significantly attenuated the number of ED-1–positive cells. Myelination in the periventricular area was also evaluated on P32 by determining the optical density of MBP via immunostaining. This analysis showed that myelination was significantly reduced in the IC group compared to the NC group and that this impaired myelination was markedly ameliorated in the ID group (Fig 5).

### Inflammatory cytokines in the brain

**Fig 6:**
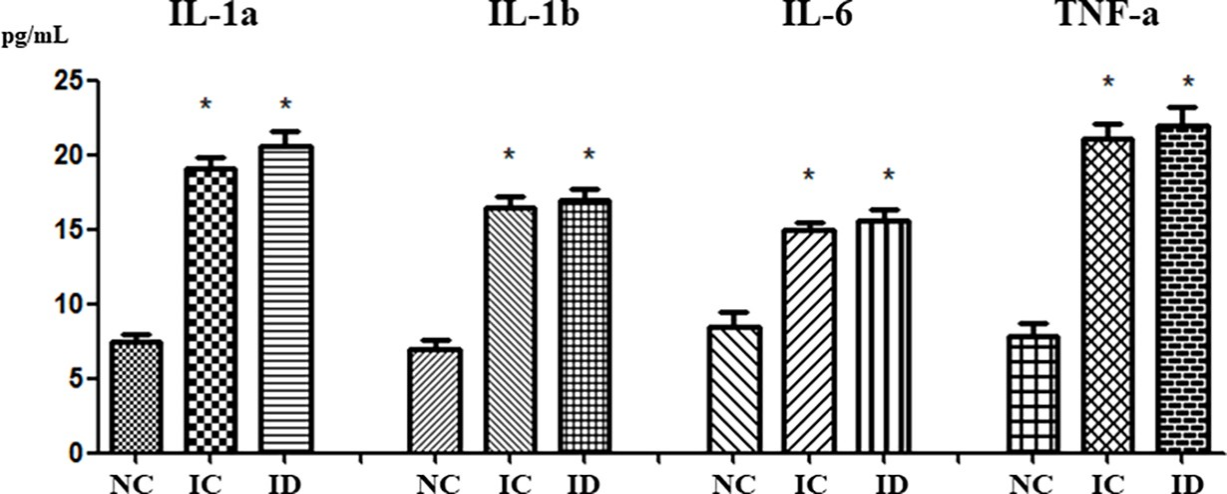
The concentrations of the pro-inflammatory cytokines (interleukin [IL]-1α, IL-β, IL-6, and tumor necrosis factor [TNF]-α) in brain homogenate taken from the periventricular area on day P32. The numbers are expressed as the mean ± SEM. IVH refers to intraventricular hemorrhage. *P < .05 compared to the normal control (NC, n = 5). IC, IVH control (n = 10); ID, IVH + dexamethasone (n = 11).

To determine whether dexamethasone attenuated brain inflammation induced by severe IVH, we analyzed inflammatory cytokine concentrations, including IL-1α, IL-1β, IL-6, and TNF-α in periventricular brain tissue homogenates on P32. The IC group had greater cytokine levels than the NC group, but dexamethasone did not attenuate the levels of these cytokines significantly (Fig 6).

## Discussion

Despite recent advances in neonatal intensive care medicine, severe IVH and the ensuing PHH remain serious diseases with high mortalities and neurologic morbidities in survivors [27]. Currently, few effective therapies are clinically available to improve the prognosis of this intractable and devastating neonatal disorder. Therefore, the development of an appropriate animal model to simulate clinically severe IVH and ensuing PHH is an essential first step in determining its pathophysiological mechanisms, and to test the therapeutic efficacy of any potential new treatments. In the present study, we used P4 rats, whose brains are comparable in maturation to a human brain at approximately 27 weeks gestational age [28], because severe IVH is more common in less mature infants [29]. A large rat litter size provided a sufficient number of rat pups from one litter to use in the experimental induction of severe IVH, and their larger size compared with mice enabled easier surgical manipulation at an earlier age along with a larger amount of brain tissue that could be harvested. We have developed newborn rat model of severe IVH via intraventricular injection of 200 uL dam’s blood using a stereotactic frame [23] due to low induction of IVH (54%), development of post-hemorrhagic hydrocephalus (42%) and neurologic impairments (25%) with currently available GMH-IVH rabbit pup model with glycerol treatment [30]. As we have observed persistent induction of severe IVH, the progress of post-hemorrhagic hydrocephalus, brain injury and long-term neurologic impairments in the present and previous studies [23], our data, which showed the progression of severe IVH to PHH via *in vivo* brain MRI, impaired behavioral function, and histologic abnormalities indicated that this newborn rat pup model was appropriate to research the disease pathogenesis and to test the efficacy of new treatments.

As severe IVH in preterm infants is a critical disease that frequently results in mortality, the experimental design simulating this condition is inevitably subject to death due to the severity of that model itself. The use of mortality as an endpoint are somewhat opposed to an ethics viewpoint in animal experiment. However, the comparison of mortality among the groups in this study is necessary for precluding distortion of the entire experimental data. It would be helpful to develop an animal model that can utilize alternative method to assessment of mortality regarding this issue for the animal welfare.

Dexamethasone is clinically available and is often used to prevent BPD in premature infants [31]. Therefore, any favorable results from a trial of dexamethasone treatment for severe IVH could be readily translated into clinical practice. In this study, we have chosen the same tapering dose of dexamethasone starting from 0.5mg/kg that is commonly used in clinical practice to prevent BPD in the premature infants, and the same tapering dose of 0.5, 0.3 and 0.1mg/kg dose use in the newborn animal study [32].

Several studies have examined the neuroprotective effect of dexamethasone in brain injury models. In one rat model of collagenase-induced intracerebral hematoma, dexamethasone (1 mg/kg) was injected 2, 4, or 6 hours after the induction of cerebral hematoma and again after 24 hours. Improvement in neurological functions, reduction of hematomas, neutrophil infiltration, and neuronal necrosis was observed [33]. In this study, after the induction of severe IVH on P4, dexamethasone was injected at a dose of 0.5 mg/kg, 0.3 mg/kg, and 0.1 mg/kg on P5, P6, and P7, respectively. This treatment significantly ameliorated the severe IVH-induced increase in apoptosis, astrogliosis, microgliosis, and reduced myelination. In a newborn rat model of hypoxic-ischemic brain injury where dexamethasone (0.25 mg/kg) was injected 4 and 24 hours before exposure to the hypoxic- ischemic injury, a neuroprotective effect via the phosphatidylinositol 3-kinase/Akt pathway was detected [34]. A single dose of dexamethasone (0.01to 0.5 mg/kg) 24 hours before hypoxic ischemic injury in newborn rats effectively reduced cerebral infarctions [35]. Pre-treatment with dexamethasone 22 hours before hypoxic-ischemic injury preserved cerebral energy metabolism through ketogenesis in newborn rats [36]. Unlike studies that reported dexamethasone as effective at treating brain injuries, the present investigation indicated that dexamethasone only improved the associated brain injury without PHH reduction. This result can be attributed to the fact that administration of the dexamethasone occurred relatively late. It is important to note that although dexamethasone is the most extensively studied glucocorticoid with proven anti-inflammatory effects on BPD, dexamethasone treatment within one week after birth might increase the risk of neurodevelopmental disabilities and cerebral palsy [37]. Dexamethasone treatment after the first postnatal week could affect mortality rates or long-term neurodevelopmental disability [38]. Overall, conclusion from the available data about the role of dexamethasone for treating severe IVH remains largely controversial, and further research is necessary to determine the optimal timing and doses of dexamethasone that will provide maximal therapeutic effect while minimizing the inherent risks.

IVH is caused by the disturbance of cerebral blood flow in preterm infants, which disturbs a fragile, highly vascularized germinal matrix located along the lateral ventricle, causing it to rupture. Hemolysis follows the bleeding from the germinal matrix into the ventricle and results in an extracellular hemoglobin increase that is known to provoke proinflammation, chemotaxis, and apoptosis in intracranial bleeding [39, 40]. PHH development after IVH in preterm infants is associated with fibrosis in arachnoid granulations, meningeal fibrosis, and subependymal gliosis in conjunction with CSF resorption impairment [41]. It has been suggested that inflammation can induce PHH progression from IVH in preterm infants by causing fibrosing arachnoiditis, meningeal fibrosis, and subependymal gliosis [41, 42] and that inflammation also causes secondary brain injury, such as neuronal death and reactive gliosis after IVH [43–45]. Moreover, Savman et al. reported that preterm infants with PHH after IVH had increased levels of pro-inflammatory cytokines, such as TNF-a, in the CSF [46]. In this study, rat pups treated with dexamethasone experienced some neuroprotective effects, including ameliorations in severe IVH induced-increases in TUNEL-positive and ED-1 positive cells and improvements in IVH-induced, reduced MBP, along with a tendency to improve survival from 71% to 86%. However, dexamethasone treatment did not significantly downregulate the increased levels of inflammatory cytokines such as IL-1α, IL-1ß, IL-6, and TNF-α, and thereby failed to attenuate the progress of PHH after severe IVH. In the developing brain, there is a vulnerable time window of reduced anti-oxidant system and increased TUNEL positive cells [47] and at the highest risk for impaired myelination and the ensuing periventricular leukomalacia [48]. Therefore, our data of significantly improved brain myelination and significantly reduced apoptotic cell death, astrogliosis and microglial cells suggest that dexamethasone treatment started at 0.5 mg/kg dose at P5 falls within the therapeutic time window, and effective enough to significantly attenuate severe IVH induced brain injury. In our previous studies, MSCs transplantation given at P6 but not at P11 significantly attenuated severe IVH induced progress of ventriculomegaly and brain injury [49], and antenatal betamethasone treatment significantly attenuated intrauterine infection induced lung injuries but not the ongoing postnatal hyperoxia induced lung injuries [50]. These findings suggest that besides optimal dosage, timing and duration are also important determinant of therapeutic efficacy of dexamethasone treatment. Collectively, our data of improvement of TUNEL/GFAP/ED-1 without decrease in pro-inflammatory cytokines and improved post-hemorrhagic hydrocephalus might be attributable to lack of sustained attenuation of severe IVH induced ongoing inflammation despite its initial therapeutic efficacy with tapering doses of 0.5, 0.3, and 0.1 mg/kg dexamethasone treatment. Further studies will be necessary to clarify this.

Unfortunately, our data that are in direct contrast to the classical anti-inflammatory responses expected after dexamethasone treatment [51] remain difficult to explain. Similarly, in male Sprague-Dawley rats, 0.1–10 mg/kg dexamethasone pretreatment aggravated the hyperoxic lung inflammatory responses [52], and chronic glucocorticoid exposure facilitated paradoxical pro-inflammatory responses to injury in the brain [53, 54]. Overall, these findings suggest that the classification of dexamethasone as a strict anti-inflammatory agent might be an oversimplification and that further studies are necessary to elucidate the mechanism of the contradictory pro- and anti-inflammatory effects of dexamethasone for its future clinical use with optimal efficacy and safety.

In order for dexamethasone treatment for severe neonatal IVH to be translated into clinical practice, both improved histologic findings and improved functional outcomes are needed. In this study, dexamethasone treatment ameliorated severe IVH-induced increases in apoptosis, astrogliosis, and microgliosis, and improved severe IVH-induced decreases in myelination. However, dexamethasone treatment failed to attenuate the inflammatory responses, prevent the progression of PHH, or improve the impaired neurobehavioral tests such as the negative geotaxis test or rotarod test. These findings suggest that inflammatory responses and the progression of PHH are more important than improved myelination, apoptosis and astrogliosis for improved behavioral function tests after severe IVH.

In summary, despite improved histologic findings such as reduced apoptosis, astrogliosis and microgliosis, and increased myelination, dexamethasone failed to improve inflammatory responses, the progression of PHH, or impaired behavioral function tests after severe IVH in newborn rats.
